# *High-level expression of sugar inducible gene2* (*HSI2*) is a negative regulator of drought stress tolerance in *Arabidopsis*

**DOI:** 10.1186/1471-2229-13-170

**Published:** 2013-10-29

**Authors:** Nirmala Sharma, Yarnel Bender, Kerry Boyle, Pierre R Fobert

**Affiliations:** 1 National Research Council Canada, 110 Gymnasium Place, Saskatoon, SK S7N 0W9, Canada

**Keywords:** Abiotic stress, Abscisic acid, Gene expression, Microarray, Transcriptome, Water stress

## Abstract

**Background:**

*HIGH-LEVEL EXPRESSION OF SUGAR INDUCIBLE GENE2* (*HSI2*), also known as *VAL1*, is a B3 domain transcriptional repressor that acts redundantly with its closest relative, *HSI2-LIKE1* (*HSL1*), to suppress the seed maturation program following germination. Mutant *hsi2 hsl1* seedlings are arrested early in development and differentially express a number of abiotic stress-related genes. To test the potential requirement for HSI2 during abiotic stress, *hsi2* single mutants and plants overexpressing *HSI2* were subjected to simulated drought stress by withholding watering, and characterized through physiological, metabolic and gene expression studies.

**Results:**

The *hsi2* mutants demonstrated reduced wilting and maintained higher relative water content than wild-type after withholding watering, while the overexpressing lines displayed the opposite phenotype. The *hsi2* mutant displayed lower constitutive and ABA-induced stomatal conductance than wild-type and accumulated lower levels of ABA metabolites and several osmolytes and osmoprotectants following water withdrawal. Microarray comparisons between wild-type and the *hsi2* mutant revealed that steady-state levels of numerous stress-induced genes were up-regulated in the mutant in the absence of stress but down-regulated at visible wilting. Plants with altered levels of *HSI2* responded to exogenous application of ABA and a long-lived ABA analog, but the *hsi2* mutant did not show altered expression of several ABA-responsive or ABA signalling genes 4 hr after application.

**Conclusions:**

These results implicate HSI2 as a negative regulator of drought stress response in *Arabidopsis*, acting, at least in part, by regulating transpirational water loss. Metabolic and global transcript profiling comparisons of the *hsi2* mutant and wild-type plants do not support a model whereby the greater drought tolerance observed in the *hsi2* mutant is conferred by the accumulation of known osmolytes and osmoprotectants. Instead, data are consistent with mutants experiencing a relatively milder dehydration stress following water withdrawal.

## Background

Water deficit is a major environmental factor limiting plant fitness and productivity [1]. With patterns of global climate change likely to increase the severity of drought stresses in the future [2], the development of crop plants better adapted to water-limited environments is a priority for sustainable agriculture.

Plants have evolved unique mechanisms to monitor water availability in their environment and adapt to it [3,4]. In response to mild water deficits, low cellular water potential can be avoided by balancing water uptake and loss through stomatal closure, increased cuticle thickness and enhanced root growth. Should these adaptations be insufficient to restore physiological water potential within the cell, more dramatic measures may be deployed. These are generally distinguished as dehydration avoidance, dehydration tolerance and drought escape [3], although all three responses are highly integrated [5]. A key aspect of dehydration avoidance is the accumulation of compatible solutes (osmolytes), including sugars, sugar alcohols, amino acids and organic acids, that increase cellular osmotic potential, prevent water loss and maintain turgor [6]. Dehydration tolerance consists of measures to protect cell constituents from damage and entails the production of antioxidants and chaperones, such as dehydrins and late-embryogenesis abundant (LEA) proteins [7].

The plant hormone abscisic acid (ABA) plays a pivotal role in coordinating multiple aspects of adaptation to drought stress [8]. Stomatal closure, the accumulation of osmolytes and the synthesis of protective proteins are all correlated with drought-induced increases in endogenous ABA levels. Mutants defective in ABA synthesis or perception are more susceptible to drought, while treatment with exogenous ABA enhances drought tolerance and induces the expression of numerous dehydration-stress responsive genes and proteins. However, several dehydration stress-induced genes do not respond to exogenous application of ABA in *Arabidopsis thaliana* (L.) [9,10], suggesting the existence of both ABA-dependent and -independent signal transduction pathways.

Physiological and biochemical aspects of drought adaptation are underpinned by extensive transcriptional re-programming [9]. Drought-induced transcriptomes from several plant species have been reported (reviewed in [11]) and reveal profound changes in numerous processes, including growth, amino acid and carbohydrate metabolism, photosynthesis, protection against oxidative stress, phosphorylation, membrane transport, secretion, cell wall expansion, and hormone homeostasis [10,12-15]. Functional characterization efforts have identified the role of a number of drought-induced transcripts, including many transcription factors, in driving adaptive responses [10,16-18]. Differential gene expression in response to abiotic stress is likely associated with changes in chromatin conformation, such as those mediated by histone tail modifications and chromatin remodeling complexes [19,20]. Identification and characterization of the factors and mechanisms involved is only now being elucidated and an active area of research.

One group of proteins that may be implicated in regulating plant chromatin conformation is the HIGH-LEVEL EXPRESSION OF SUGAR-INDUCIBLE GENE2 (HSI2) clade of B3 domain proteins. The B3 domain is a plant-specific basic DNA-binding domain originally identified as the third and C-terminus proximal basic domain of the transcription factor ABSICIC ACID INSENSITIVE3 (ABI3) (reviewed in [21]). There are three members of the HSI2 clade in *Arabidopsis*; HSI2, also known as the VIVIPAROUS ABI3-LIKE1 (VAL1), HSI2-LIKE1 (HSL1*,* also known as VAL2) and HSI2-LIKE2 (HSL2*,* also known as VAL3) [22-24]. Additional features shared by HSI2 clade proteins include the CW domain and EAR motif. HSI2 and HSL1 also contain a putative plant homeodomain (PHD)-like zinc finger domain absent from HSL2. The CW and PHD-like domains are associated with chromatin remodelling factors, while the EAR motif is a transcriptional repression domain. Functional analysis of HSI2 has demonstrated that the protein is a potent EAR-dependent transcriptional repressor [23] and can repress ABI3-mediated transactivation from the Sph/RY element, the cognate DNA binding site of B3 domain factors [25]. Loss-of-function mutations in *HSI2* result in the expression of seed storage proteins in vegetative tissues [23,24]. The deregulation of embryonic and seed maturation programs in vegetative tissues is more dramatic in *hsi2 hsl1* (*val1 val2*) double mutants, which produce callus and ectopic embryo-like structures when seedlings are cultured in the presence of sucrose. Among genes highly expressed in the double mutant seedlings are *ABI3*, *FUSCA3* (*FUS3*) and *LEAFY COTYLEDON2* (*LEC2*), which encode key regulators of seed maturation and constitute the AFL (ABI3/FUS3/LEC2) clade of B3 domain proteins. It has been proposed that HSI2 clade proteins suppress the seed maturation program following germination by repressing the transcription of AFL clade genes and/or competing with the encoded transcription factors for Sph/RY sites in the promoter of target genes [22,24,25].

The recent analysis of the *hsi2-4* allele, which creates a cysteine to tyrosine substitution in the PHD domain, revealed a potential role for this HSI2 domain in the repression of a subset of seed-specific genes during seedling development and in the deposition of the repressive chromatin mark H3K27m3 on target genes [26]. Interestingly, *hsi2-4 hsl* double mutants were morphologically normal, indicating a unique function for the HSI2 PHD domain.

Molecular genetic analysis of the HSI2 clade has focused on *hsi2 hsl1* double mutants at the seedling stage. The potential role of these factors at other times in the plant life cycle remains largely unexplored, despite the fact that *HSI2* and *HSL1* are expressed in many tissues [23]. A cursory analysis of gene ontology (GO) annotation terms enriched in the list of genes differentially expressed in *hsi2* and *hsi2 hsl1* mutants [24,26] identified response to abiotic stimulus as highly significant (data not shown). This prompted us to test whether *HSI2* might regulate drought tolerance during the vegetative stage of the plant life cycle. Through loss- and gain-of-function analyses, *HSI2* is shown to act as a negative regulator of drought tolerance in *Arabidopsis*, possibly through a mechanism involving reduced transpirational water loss.

## Results

**Figure 1:**
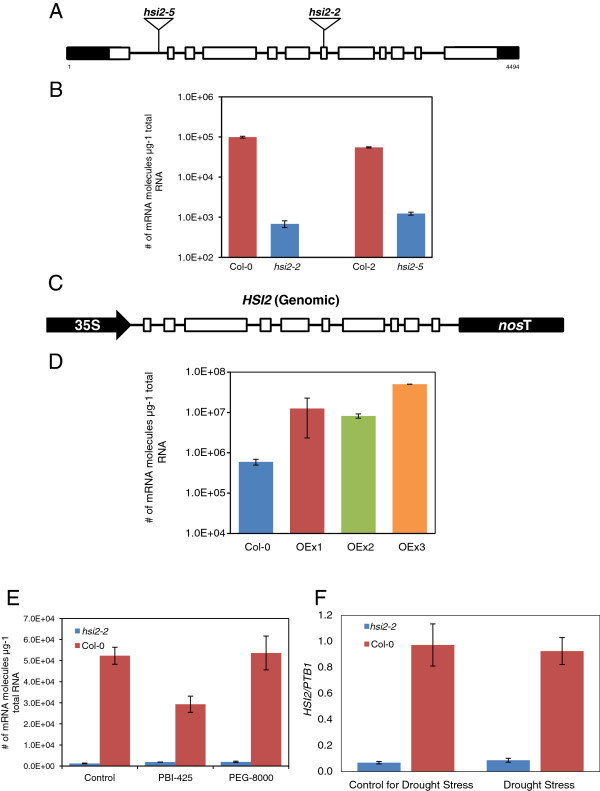
***HSI2 *insertional mutants and overexpressing lines. (A)** Diagram of the structure of the *HSI2* gene (At2g30470) showing the position of T-DNA insertions (triangles). Open boxes indicate exons, while black boxes indicate untranslated regions. The transcriptional start site is indicated by the number 1. With the exception of T-DNA sizes, the diagram is drawn to scale. **(B)** Kinetic RT-PCR analysis of *HSI2* mRNA abundance in the mutants and corresponding wild-types (*hsi2-2/*Col-0 and *hsi2-5*/Col-2). **(C)** Diagram of the structure of the *HSI2* transgene. Open boxes indicate exons; 35S, Cauliflower mosaic virus 35S promoter; *nos*T, terminator region from the *Agrobacterium tumefaciens nopaline synthase* gene. **(D)** Kinetic RT-PCR analysis of *HSI2* mRNA abundance in transgenic plants and untransformed Col-0. **(E)** Kinetic RT-PCR analysis of *HSI2* mRNA abundance in 14 day old seedlings of *hsi2-2* and Col-0, 4 h following treatment with 25 μM PBI425 or 20% PEG 8000. **(F)** Kinetic RT-PCR analysis of *HSI2* mRNA abundance in 3-week-old leaves of *hsi2-2* and Col-0 grown in soil saturated to field capacity or following watering withdrawal and development of visible wilting symptoms. For **(F)**, amplification of the housekeeping gene encoding polypyrimidine tract-binding protein1 (PTB1, AT3G01150) was used as control to normalize expression data. All values represent the averages of three biological replicates, each analyzed three times (technical replicates) ± standard error.

To investigate the role of *HSI2* in modulating drought responses, the levels and/or integrity of the gene were stably altered in transgenic *Arabidopsis* plants. For loss-of-function analysis, two T-DNA insertion alleles were identified (Figure 1A). Both mutant alleles show minimal expression of *HSI2* when compared to corresponding wild-types, Col-0 for *hsi2-2* and Col-2 for *hsi2-5* (Figure 1B). For gain-of-function analysis, the *HSI2* coding region was placed under the control of the Cauliflower mosaic virus 35S promoter (*35S:HSI2*) and used to generate transgenic plants in Col-0 (Figure 1C). Three independent lines showing increased levels of *HSI2* transcripts (Figure 1D, OEx lines) were further studied.

### Disruption of *HSI2* by T-DNA insertions confers better drought tolerance

**Figure 2:**
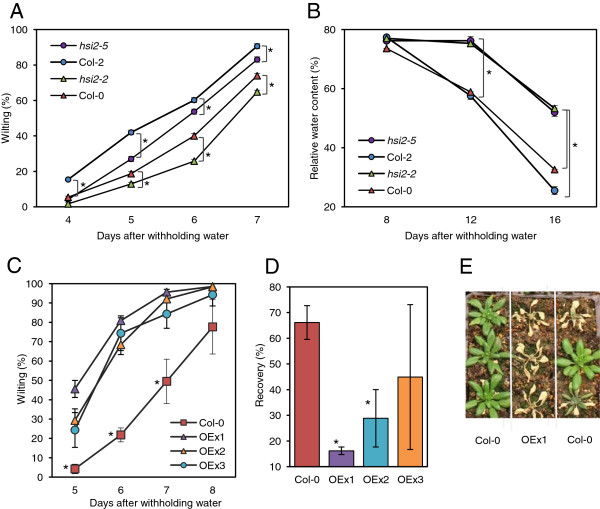
**Response of *hsi2 *mutants and *35S:HSI2 *overexpressing lines to simulated drought stress. (A)** Wilting rates of *hsi2* mutants and corresponding wild-types. **(B)** Leaf relative water content of the *hsi2* mutants and corresponding wild-types. **(C)** Rate of wilting of three independent *35S:HSI2* lines and untransformed wild-type (Col-0). **(D)** Recovery from drought 24 h upon re-watering. **(E)** A representative photograph showing the recovery phenotype of Col-0 and *35S:HSI2* line OEx1. For **(A)** and **(B)**, the *hsi2-2* mutant compares with Col-0 and *hsi2-5* compares with Col-2; each wild-type and mutant combination were planted in the same sets of containers. Statistical significance, indicated by an asterisk(*), was determined by a paired Student’s *t*-test (p ≤ 0.05). Data are presented as means of three replicates containing 12 plants each ± standard error of means. In some cases where the standard errors are very small, the graph symbols may obscure the bars. Experiments were repeated at least three times with similar results.

The response of *hsi2* mutants to drought was examined by subjecting plants to a simulated drought stress regime in environmentally controlled growth chambers. To minimize sources of variation attributed to the amount, and the initial moisture content, of the soil in pots, wild-type and corresponding mutant plants were grown side-by-side in the same containers. Both *hsi2* mutants displayed reduced rates of wilting after withholding water, by as much as 9%, compared to their wild-type counterparts (Figure 2A). The *hsi2* mutants also maintained higher cellular water levels, by as much as 30%, measured as leaf relative water content (RWC; Figure 2B). Additional experiments using different pot sizes and potting mixes yielded similar findings (data not shown). These results indicate that *hsi2* mutants have increased tolerance to drought, due at least in part to a reduced rate of water loss from leaves.

### *HSI2* overexpression enhances drought sensitivity

The three independent OEx lines evaluated showed an early onset and greater severity of visual wilting, and poorer recovery from a drought stress compared to untransformed controls (Figure 2C-E). Differences in visible wilting were most pronounced at 6 and 7 days after withholding water, with OEx lines displaying between 48-60% and 35-45% higher wilting rates, respectively (Figure 2C). When plants were re-watered at the end of the drought period and allowed to recover for 24 h, 66% of the wild-type plants recovered compared to 16, 24, and 44% in the OEx lines (Figure 2D, 2E). Together with data obtained from loss-of-function mutants, the results implicate *HSI2* as a negative regulator of drought tolerance.

### *HSI2* altered plants respond to an exogenously applied ABA

To study the possible interaction between *HSI2* and ABA during drought stress, we assessed the drought response of the *hsi2-2* mutant and line OEx1 pre-treated with (+)-ABA and (+)-8’-acetylene ABA, also known as PBI425 [27]. This synthetic analog is resistant to 8’hydroxylation, the principal ABA catabolic pathway in *Arabidopsis*[12,28]. Consequently, PBI425 is more persistent *in planta* and has stronger hormonal activity [29,30]. The ability of PBI425 to enhance drought tolerance in *Arabidopsis* has been shown to be mediated through ABA signalling [29]. Whether PBI425 and ABA act through the same signaling pathway can be tested by binding to the family of PYL receptors. Nevertheless, available information indicate that PBI425 offers several advantages over natural ABA when the sustained presence of hormonal activity is required, as is the case in this study.

**Figure 3:**
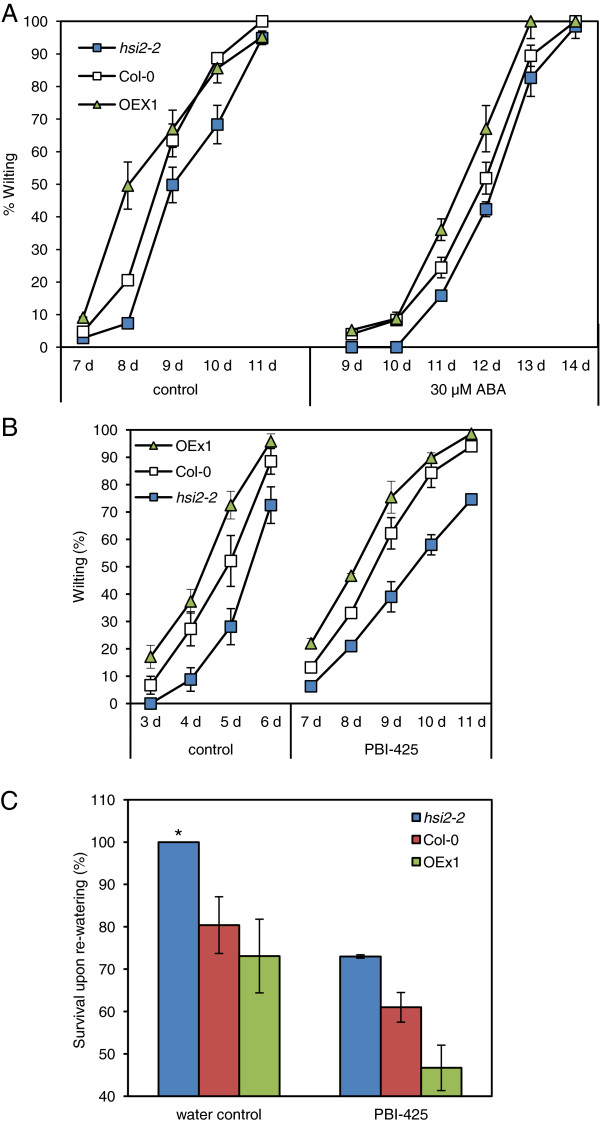
**Response of *hsi2 *mutants and *35S:HSI2 *overexpressing lines to simulated drought stress. (A-B)** Rate of wilting of wild-type (Col-0), mutant (*hsi2-2*), and *35S:HSI2* line (OEx1) treated with (+)-ABA **(A)** or the ABA analog PBI425 **(B)**. **(C)** Recovery from drought upon re-watering (at 24 h after re-watering under control and PBI425 treatments). Three-week-old plants were root drenched with 0.05% ethanol as control treatment (left side), 30 μM ABA or 20 μM PBI425, dissolved in 0.05% ethanol (right side) to field capacity and watering was stopped thereafter. Data are presented as means of three replicates of 18 plants each ± standard error of means. In some cases where the standard errors are very small, the graph symbols may obscure the bars. These experiments were repeated at least twice with similar results.

In control treatments where plants were root-drenched with water prior to water withdrawal, *hsi2-2* plants displayed less wilting, and OEx1 plants more wilting, than the wild-type at all observation times (Figure 3A and 3B). When plants were given a root-drench treatment with ABA (30 μM) or PBI425 (20 μM) before withholding water, the onset of visible wilting was delayed in all genotypes by three to four days (until 7 – 10 days after treatment), after which the mutant continued to display less wilting, and OEx1 more wilting, than the wild-type at all observation times (Figure 3A and 3B). Recovery from wilting upon re-watering was evaluated under control and ABA analog treatments, where the *hsi2-2* mutant recovered the best upon re-watering, followed by the wild-type and OEx1 plants (Figure 3C). However, the overall recovery upon re-watering was lower in all genotypes pre-treated with PBI425.

### *Hsi2-2* mutants display lower stomatal conductance

**Figure 4:**
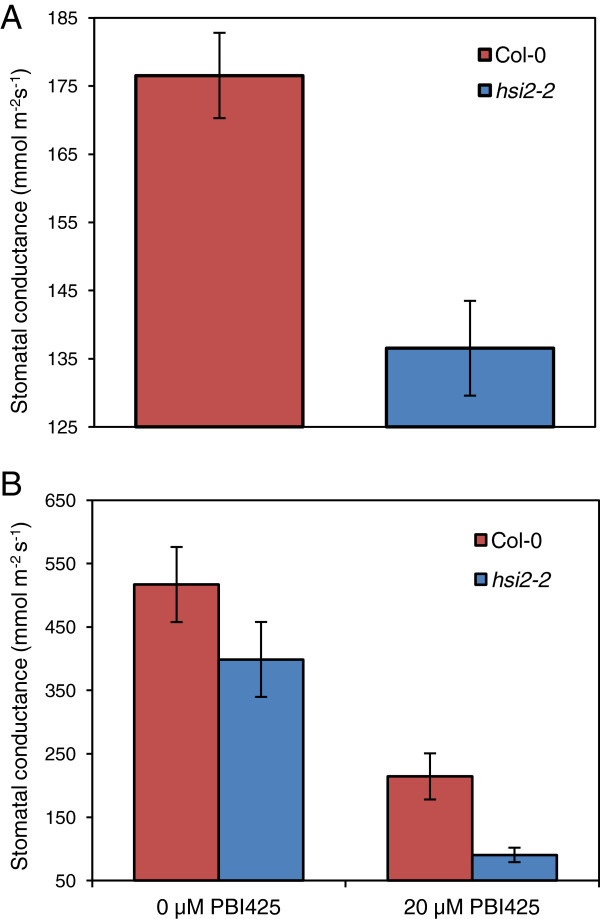
**Stomatal conductance in the *hsi2-2 *mutant and corresponding wild-type (Col-0).** Stomatal conductance was measured in fully developed young leaves **(A)** 1 d after watering or **(B)** 72 h after root drench treatment with 0 or 20 μM PBI425 (in 0.05% ethanol). Data presented as means of three replicates ± standard error of means, each containing 4–6 plants and measurements from two leaves per plant. The experiments were repeated twice with similar results.

Lower stomatal conductance has been associated with higher drought tolerance in many plant species including *Arabidopsis*[31,32]. Accordingly, stomatal conductance was measured to investigate possible mechanisms underpinning the increased drought tolerance in the *hsi2* mutant. Leaf stomatal conductance in well-watered *hsi2-2* plants was 77% of wild-type values (Figure 4A), suggesting lower constitutive stomatal conductance. Pre-treatment with PBI425 resulted in a 2.4-fold reduction in stomatal conductance in the wild-type and a 4.4-fold reduction in *hsi2-2* (Figure 4B), with the *hsi2-2* mutant displaying 42% of wild-type stomatal conductance following pre-treatment with PBI425 (Figure 4B). These results suggest that the mutant is able to better maintain water status through regulation of stomatal properties and that loss of *HSI2* does not substantially affect associated ABA signaling.

### The *hsi2-2* mutant accumulates less ABA metabolites during drought stress

**Figure 5:**
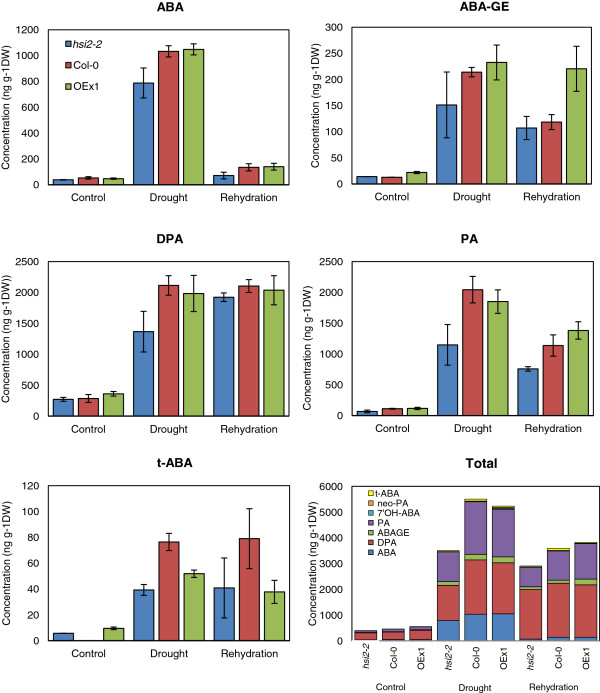
**Levels of ABA and its metabolites in leaves of *HSI2 *altered Arabidopsis plants.** Levels were quantified by UPLC-ESI-MS/MS using deuterium-labeled internal standards. Each sample consisted of a pool of 6 plants grown under well-watered (to near field capacity) or drought-stressed (visible wilting) conditions, and 24 h after re-watering to soil saturation following drought stress. Values represent averages of three biological replicates ± standard error of means. No error bars are provided in the Total ABA panel. DPA, dihydrophaseic acid; ABAGE, ABA glucose ester; 7’-OH ABA; PA, phaseic acid; t-ABA, *trans*-ABA, OEx1, *35S:HSI2* line.

To examine the involvement of *HSI2* in ABA metabolism during the drought response, levels of endogenous ABA and its metabolites were measured in leaves of *hsi2-2*, OEx1*,* and Col-0 before drought stress, at visible wilting, and upon rehydration. In the absence of drought, levels of all metabolites were similar in the three genotypes (Figure 5). Endogenous ABA content increased dramatically under drought stress in all three genotypes, and reverted to near pre-stress levels upon re-watering. However, *hsi2-2* accumulated relatively lower levels of ABA during drought and was better able to recover to original levels after re-watering compared to OEx1 or Col-0. Levels of ABA metabolites, namely ABA-glucose ester (ABA-GE), dihydrophaseic acid (DPA), phaseic acid (PA), and *trans*-ABA (*t*-ABA) also increased substantially under drought stress in all genotypes (Figure 5). The *hsi2-2* mutant accumulated less of these metabolites than Col-0, while OEx1 contained levels comparable to Col-0, with the exception of *t*-ABA, which was lower in *35S:HSI2*. Consistent with the reports that the 8’-hydroxylation pathway is the most important ABA catabolic pathway in *Arabidopsis*[12,28], the products of this pathway (PA and DPA), were the most abundant during drought stress and after rehydration (Figure 5). Unlike ABA, the levels of most metabolites remained much higher after rehydration than they were pre-drought, with DPA levels remaining as high as during drought stress. These results indicate that drought-induced ABA biosynthesis and catabolism is of smaller amplitude in the mutant line, resulting in a more modest increase in both ABA and ABA metabolites, while rehydration reversed the effect of drought on ABA biosynthesis but not ABA catabolism.

### The *hsi2-2* mutant accumulates lower levels of stress-induced metabolites during drought stress

**Figure 6:**
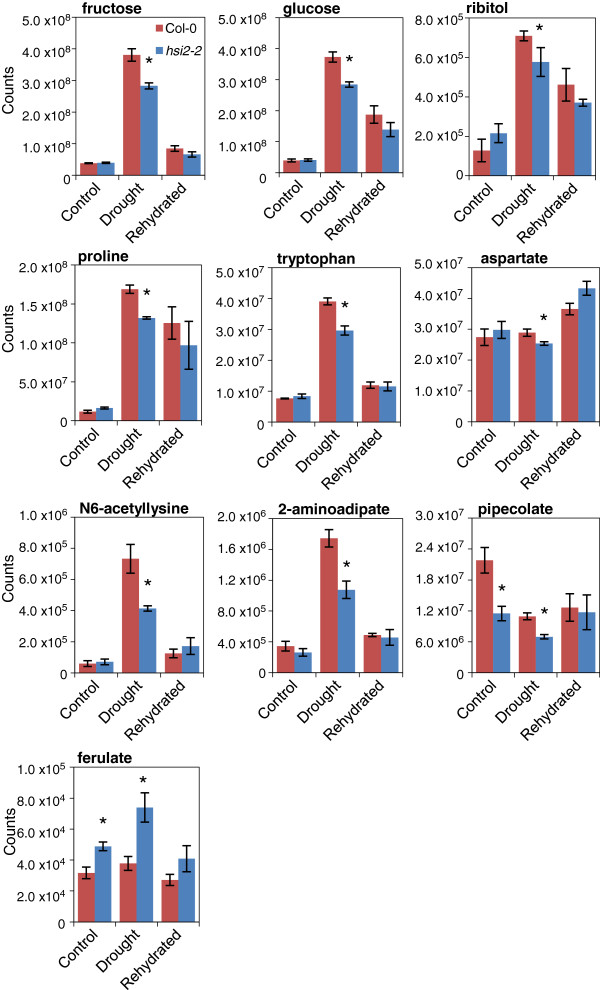
**Drought-related levels of selected metabolites showing significant changes in leaves of the *hsi2-2 *mutant*.*** Each sample consisted of a minimum of 6 plants grown under well-watered (to near field capacity) or drought-stressed (visible wilting) conditions, and 24 h after re-watering to soil saturation following drought stress. Values represent averages of three biological replicates ± standard error. Treatments statistically different by ANOVA (p ≤ 0.05) are marked by asterisks.

To ascertain the role of *HSI2* during stress-induced metabolic reprogramming, levels of several compounds were measured by undirected metabolic profiling and compared between *hsi2-2* and Col-0 before drought stress, at visible wilting, and upon rehydration. Overall, drought treatment increased levels of numerous metabolites previously reported to be drought-inducible [33], including soluble sugars, sugar alcohols, amino acids, organic acids and antioxidants (Figure 6; Additional file 1). Levels of several metabolites were also depleted under drought conditions. With some exceptions, most metabolites reverted closer to non-stress levels upon rehydration.

Relatively few of the stress-regulated metabolites were found to accumulate differentially in the two genotypes tested (Figure 6; Additional file 1). These included sugars (fructose and glucose), amino acids (proline, tryptophan and aspartate), and the sugar alcohol ribitol, levels of which were lower in the mutant than the wild-type under drought stress. Similarly, the products of the lysine degradation pathway, N6-acetyllysine, 2-aminoadipate and pipecolate, which have been shown to accumulate under osmotic stress in *Brassica napus*[34] and suggested to have osmoprotectant properties in bacteria (pipecolate [35]) were also lower in the mutant compared to wild-type under drought conditions. In contrast, *hsi2-2* had significantly higher levels of ferulate, an intermediate of the phenylpropanoid pathway, before and during drought (Figure 6). Lower levels of known stress-induced osmolytes and osmoprotectants in the *hsi2* mutant at wilting are consistent with plants experiencing relatively milder dehydration stress than the wild-type.

### Analysis of drought transcriptomes indicates that drought-induced genes are down-regulated in the *hsi2* mutant

**Figure 7:**
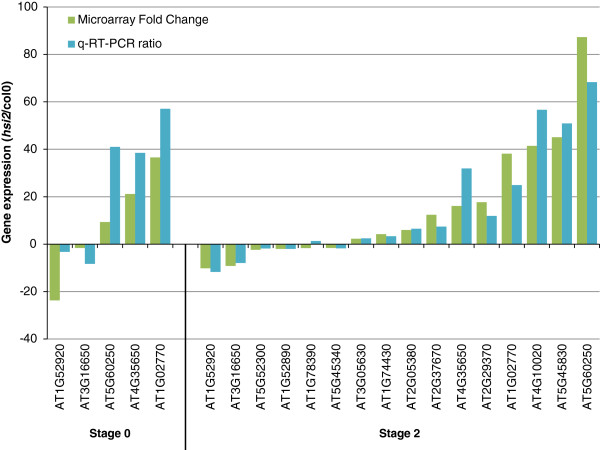
**Kinetic RT-PCR validation of microarray analyses for a representative subset of genes.** cDNA was synthesized from the areal portion of well watered plants (Stage 0) and at visible drought (Stage 2). Amplification of the housekeeping gene encoding polypyrimidine tract-binding protein1 (PTBpa, AT3G01150) was used as control to normalize PCR expression data. All values represent the averages of three biological replicates, each analyzed three times (technical replicates). Values from microarray expression are ratios of averaged, normalized fluorescent signals.

To characterize the consequences of loss of *HSI2* function on gene expression relevant to drought stress, genome-wide expression analysis was undertaken with the *hsi2-2* mutant under a normal watering regime (no stress; stage 0) and after withholding watering, before the onset of visible wilting (stage 1) and at visible wilting (stage 2). Gene expression in the mutant was compared with its wild-type (Col-0) under each watering regime by co-hybridization using dual color microarrays. Genes corresponding to 441 unique AGIs were identified as being differentially expressed (*p* ≤ 0.05, 1.5-fold cut-off) at stage 0, and the numbers of differentially expressed unique AGIs increased with the severity of drought to 785 (stage 1) and 1400 (stage 2) (Additional files 2 and 3). Consistent with differences in drought responses observed between the mutant and wild-type (Figures 2, 3, 4), the most significant GO enriched terms for biological process identified in lists of differentially expressed genes were response to stress and response to biotic or abiotic stimulus (Table 1). The relative *p*-values for these terms decreased with the severity of the drought treatment. The identity of genes differentially regulated under the three watering regimes changed considerably. Of the 441 genes differentially expressed at stage 0, 335 (76%) are specific to this stage and only 48 genes were differentially regulated under all watering regimes. For representative genes, microarray results were validated by kinetic reverse-transcriptase PCR (k-RT-PCR) (Figure 7).

**Table 1:** **Gene Ontology classification terms enriched in the *hsi2-2 *** **mutant compared to Col-0 under different simulated drought regimes**

		**Stage 0**			**Stage 1**			**Stage 2**	
	**Genes**	**Enrich.**	***p*-value**	**Genes**	**Enrich.**	***p*-value**	**Genes**	**Enrich.**	***p*-value**
***Biological Process***									
unknown biological processes	160	9.3	**1.18E-04**	260	12.5	**2.67E-11**	456	16.9	**4.61E-21**
other cellular processes	140	9.7	**0.029**	261	12.8	**4.01E-03**	480	16.5	**5.74E-05**
other metabolic processes	138	10.4	**0.015**	269	13.1	**2.93E-05**	477	18.3	**2.82E-07**
protein metabolism	47	6.7	**0.042**	80	8.6	**0.013**	161	11.9	**0.028**
response to stress	40	6.3	**9.20E-04**	72	8.8	**1.48E-05**	125	9.1	**1.11E-07**
developmental processes	31	5	**0.013**	53	6.6	**5.39E-03**	86	9	**7.36E-03**
transcription	31	5.3	**0.019**	52	6.1	**0.013**	78	8.4	**0.04**
transport	28	5.2	0.051	64	8.1	**2.31E-04**	105	8.8	**1.20E-04**
response to abiotic or biotic stimulus	26	5.3	0.055	61	8.3	**1.68E-04**	114	9.5	**8.32E-08**
signal transduction	25	4.9	**3.77E-03**	35	5.2	**0.018**	65	7.7	**1.96E-03**
other biological processes	23	4.2	0.082	58	7.1	**5.15E-04**	101	10.9	**2.92E-05**
cell organization and biogenesis	15	3.6	0.091	22	5.2	**0.022**	56	7	0.054
electron transport or energy pathways	4	1.7	0.197	5	2.2	0.118	11	2.9	0.103
DNA or RNA metabolism	1	0.8	0.05	8	2.8	0.142	21	4	**0.02**
***Molecular Function***									
unknown molecular functions	135	8.9	**9.60E-06**	231	11.5	**1.23E-10**	404	17	**7.56E-20**
other binding	76	7.5	**3.20E-03**	123	9.5	**6.42E-03**	192	11.9	**0.03**
other enzyme activity	62	7.1	**1.93E-03**	120	10.5	**1.56E-06**	197	12.7	**6.77E-07**
transferase activity	45	6.4	**6.53E-03**	66	8.1	**0.03**	155	13.2	**1.75E-07**
hydrolase activity	37	5.6	0.053	81	8.3	**7.09E-04**	131	11.4	**1.37E-03**
protein binding	36	5.4	0.069	69	7.8	**0.036**	118	10.3	**0.033**
nucleotide binding	35	6.4	**0.039**	39	6	**9.76E-03**	106	9.4	**0.015**
transcription factor activity	29	5	**0.03**	59	6.6	**7.33E-04**	96	9.4	**6.60E-04**
kinase activity	25	4.9	**0.022**	35	5.8	0.059	89	8.3	**9.94E-06**
DNA or RNA binding	25	5	**9.91E-03**	50	6.5	**8.44E-03**	103	9.6	**0.019**
other molecular functions	24	4.8	0.05	36	6.7	0.067	68	8.7	**0.038**
transporter activity	16	3.5	0.101	51	6.6	**5.99E-05**	88	8.1	**1.06E-06**
nucleic acid binding	9	2.9	**1.06E-03**	15	3.9	**6.61E-06**	44	6.4	**2.91E-04**
receptor binding or activity	7	2.9	**0.024**	8	2.5	0.087	15	3.6	**0.033**
structural molecule activity	4	1.7	0.083	8	2.9	**0.048**	19	4	0.064
***Cellular Component***									
unknown cellular components	164	10.4	**0.013**	268	12.9	**2.12E-05**	415	17	**1.48E-19**
other cellular components	79	8	**1.22E-04**	110	10.7	**0.013**	218	14.1	**3.31E-05**
other membranes	58	7.5	**3.99E-03**	83	8.4	**0.017**	189	10.8	**1.20E-06**
nucleus	35	5.4	0.07	72	7.6	0.058	119	9.9	**0.023**
other intracellular components	34	6.2	**4.18E-04**	84	8.5	**0.035**	191	11.6	**0.013**
other cytoplasmic components	32	5	**0.024**	66	7.6	**0.037**	143	10.5	**0.017**
chloroplast	28	4	**4.61E-03**	66	7.6	**0.015**	152	10.9	**7.94E-03**
plasma membrane	21	4.6	**0.038**	50	6.3	**0.03**	129	10.9	**2.07E-06**
cell wall	10	2.9	0.083	13	3.6	0.104	32	5	**0.017**
cytosol	10	3.6	0.127	19	4.4	**9.12E-04**	34	6	0.061
plastid	7	2.7	**0.012**	12	3.3	0.086	45	6.9	0.061
mitochondria	6	2.6	**4.68E-03**	19	4.3	0.112	46	6.1	0.06
extracellular	6	2.5	0.163	12	3.5	**0.027**	34	5.2	**3.21E-04**
ER	4	1.9	0.177	10	2.5	0.057	15	3.8	0.102
ribosome	2	1.1	**0.035**	7	3.4	0.119	18	3.9	0.084
Golgi apparatus	1	0.7	0.142	4	1.7	0.159	11	3.2	0.114

Enrichments were performed at http://bar.utoronto.ca/ntools/cgi-bin/ntools_classification_superviewer.cgi. Statistically significant values (*p* < 0.05) are in bold. Stage 0, watered to field capacity; Stage 1, dry soil, no visible wilting; Stage 2, visible wilting.

Genes differentially expressed in the *hsi2* mutant were compared with those regulated in response to drought stress. Given the poor overlap of genes reported as being differentially expressed during drought stress in different studies (see [11,12,36]), genes regulated by drought in our datasets were identified by comparing probe intensity values from individual channels between stage 2 and stage 0 in Col-0. The direction of change for these genes in our datasets was overwhelmingly the same as those from published studies employing whole genome microarrays and similar drought regimes (Additional file 3).

Genes found to be induced in response to drought stress in our dataset (>2.5-fold) were predominantly down-regulated when differentially expressed in the *hsi2* mutant (vs. Col-0) at stage 2 (>92%), while genes repressed by drought were mostly up-regulated (93%) when differentially expressed in the *hsi2* mutant at this drought stage (Additional file 3). Similar patterns were observed when genes differentially expressed in *hsi2* vs. Col-0 at stage 2 were compared to published lists of drought-regulated transcripts of Huang *et al.*[12] or the progressive drought (pDr) and ABA-induced genes of Harb *et al*. [14] (Additional file 3). Analysis of GO terms enriched in genes induced by drought and down-regulated in *hsi2* vs. Col-0 at stage 2 identified predominantly drought-associated processes, including response to abiotic stimulus, ABA, and water deprivation (Table 2). These terms were not enriched in the gene list induced by drought and up-regulated in *hsi2*.

**Table 2:** **Representative GO terms enriched in the *hsi2-2 *** **mutant versus Col-0 and regulated by drought stress**

***Description***	***GO term***	***Enrichment***	***p-value***	***Example***	***AGI***
**Induced by Drought and Down-regulated at Stage 2 ( *hsi2 *:Col-0)**					
P* response to abiotic stimulus	GO:0009628	2.03	5.27E-04	ABA receptor GCR2	At1g52920
P response to temperature stimulus	GO:0009266	3.51	3.41E-04	RAB18, RD29B	At5g66400; At5g52300
P response to light intensity	GO:0009642	8.51	5.27E-04	ZAT12	At5g59820
P response to water deprivation	GO:0009414	4.28	2.98E-03	RAB18, ANAC019	At5g66400; At1g52890
P response to abscisic acid stimulus	GO:0009737	3.51	1.18E-03	RAB18, RD29B	At5g66400; At5g52300
F* oxygen binding	GO:0019825	3.63	4.93E-04	CYP705A12, -15, -19	At5g42580; At3g20080; At3g20100
**Induced by Drought and Up-regulated at Stage 2 ( *hsi2 *:Col-0)**					
P secondary metabolism	GO:0019748	5.48	6.12E-03	4-coumarate-CoA ligase 2	At3g21240
C* plasma membrane	GO:0005886	4.98	8.54E-03	LTP2	At2g38530
**Repressed by Drought and Up-regulated at Stage 2 ( *hsi2 *:Col-0)**					
P steroid biosynthesis	GO:0006694	12.34	7.97E-03	DWARF 4	At3g50660
P carbohydrate metabolism	GO:0005975	2.22	4.50E-04	Glycosyl hydrolase 9C2, 9B8, and 17	At1g64390; At2g32990; At2g05790
P secondary metabolism	GO:0019748	2.3	9.31E-03	ESM1, CAD4	At3g14210; At3g19450
P carboxylic acid metabolism	GO:0019752	2.28	1.10E-03	LOX2	At3g45140
F water transporter activity	GO:0005372	9.73	1.52E-04	DELTA-TIP, PIP1	At3g16240; At3g61430
F carbon-oxygen lyase activity	GO:0016835	4.31	1.27E-03	α- and β-carbonic anhydrase 1	At3g52720; At3g01500
C endomembrane system	GO:0012505	1.53	1.55E-05	RKL1, LTP7	At1g48480; At2g15050
C plastid thylakoid	GO:0031976	3.18	1.57E-04	β-carbonic anhydrase 1	At3g01500
C anchored to membrane	GO:0031225	3.65	1.31E-04	TOO MANY MOUTHS	At1g80080
**Repressed by drought and Down-regulated at Stage 2 ( *hsi2 *:Col-0)**					
P DNA metabolism	GO:0006259	6.94	7.51E-04	KRYPTONITE	At5g13960
F protein binding	GO:0005515	2.64	6.11E-03	Variant in methylation 4	At1g66040
**Induced by Drought and Down-regulated at Stage 1 ( *hsi2 *:Col-0)**					
F electrochemical potential-driven transporter activity	GO:0015290	5.88	1.65E-03	GPT2, ZIFL2	At1g61800; At3g43790
**Induced by Drought and Up-regulated at Stage 1 ( *hsi2 *:Col-0)**					
P nitrogen compound biosynthesis	GO:0044271	5.07	8.23E-03	GLN1.3	At3g17820
P response to chemical stimulus	GO:0042221	2.23	7.92E-03	FIBRILLIN	At4g04020
F oxidoreductase activity	GO:0016491	2.54	8.39E-04	FERREDOXIN3	At2g27510
F protein ser/thr phosphatase activity	GO:0004722	5.43	6.47E-03	PP2CA2	At1g30220
**Repressed by Drought and Up-regulated at Stage 1 ( *hsi2 *:Col-0)**					
P cellular carbohydrate metabolism	GO:0044262	4.24	3.01E-03	Cellulose synthase-like B1	At2g32610
F hydrolase activity	GO:0016787	1.86	9.05E-03	Methyl IAA esterase	At5g58310
**Induced by Drought and Up-regulated at Stage 0 ( *hsi2 *:Col-0)**					
P phenylpropanoid metabolism	GO:0009698	19.64	5.15E-05	TT6G, CAD	At3g51240; At1g09500
P response to temperature stimulus	GO:0009266	7.17	8.46E-03	COR15B	At2g42530
P response to abiotic stimulus	GO:0009628	3.09	3.72E-03	COR15B, AGL19	At2g42530; At4g22950
F oxidoreductase activity	GO:0016491	3.56	3.15E-03	TTG6, CAD	At3g51240; At1g09500
**Repressed by Drought and Down-regulated at Stage 0 ( *hsi2 *:Col-0)**					
P protein modification	GO:0006464	6.34	7.37E-04	HDAC 18	At5g61070
F kinase activity	GO:0016301	7.34	3.75E-04	Cysteine-rich receptor-like protein kinase 4	At3g45860

* P, Biological Process; F, Biological Function; C, Cellular Component.

In cases where multiple related classifications were significant, only a subset is listed. Lists with fewer than 3 genes are not shown. Enrichments were performed at http://bioinformatics.psb.ugent.be/ATCOECIS using *p* < 0.05.

Comparisons of gene lists differentially expressed in *hsi2* vs. Col-0 at stage 2 to lists from Wilkins *et al*. [36] or the moderate drought (mDr) treatments of Harb *et al*. [14] failed to identify a clear trend between the direction of change in *hsi2* vs. Col-0 relative to drought inducibility/repression (Additional file 3). However, drought-induced genes from these moderate drought treatments, the pDr of Harb *et al.*[14], and the present study were predominantly up-regulated when differentially expressed in *hsi2* vs. Col-0 at stage 1, reaching over 80% for genes induced in mDr1 and mDr10 of Harb *et al*. [14] and up-regulated in *hsi2* vs. Col-0 at stage 1 (Additional file 3). Genes repressed by drought or ABA did not show the complimentary trend and no clear trends were identified in the stage 0 data. Notable GO terms enriched in drought-regulated genes differentially expressed in *hsi2* vs. Col-0 at stage 0 or stage 1 include response to abiotic, temperature and chemical stimuli, and oxidoreductase activity (Table 2). Taken together, these results indicate that the *hsi2* mutant constitutively expresses a number of drought- and ABA-responsive genes but under wilting conditions, the induction or repression of drought- and ABA-regulated genes is attenuated relative to the wild-type.

### Accumulation of ABA-responsive transcripts in response to the ABA analog PBI425 is not compromised in the *hsi2* mutant

**Figure 8:**
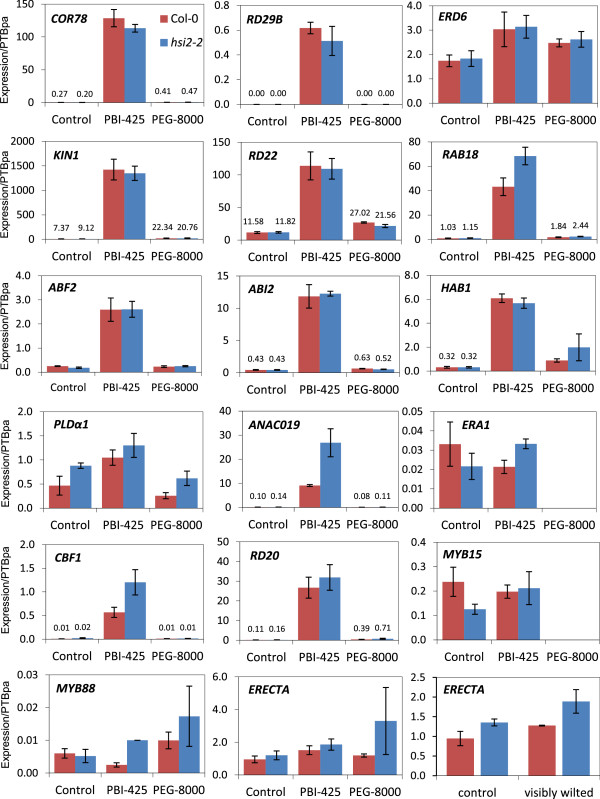
**Kinetic RT-PCR analysis of gene expression.** cDNA was synthesized from total RNA from 14-day old seedlings treated with 25 μM PBI425 or 20% PEG 8000 for 4 h. Amplification of the housekeeping gene encoding polypyrimidine tract-binding protein1 (PTBpa, AT3G01150) was used as control to normalize expression data. All values represent the averages of three biological replicates, each analyzed three times (technical replicates) ± standard error. In cases where expression levels were too small to graph, values are indicated in writing. If no bar is visible and no value indicated, the analysis was not performed.

To ascertain the possible involvement of HSI2 during the early responses to ABA and osmotic stress, RNA was extracted from two-week-old seedlings of Col-0 and *hsi2-2*, 4 h after treatment with PBI425 or PEG 8000, and the expression of selected genes was analyzed by k-RT-PCR. Steady-state mRNA levels of five well-known ABA responsive marker genes (*COR78/RD29A, RD29B*, *ERD6*, *KIN1*, and *RD22*[14,15]) continued to show wild-type induction to PBI425 and PEG 8000 in the *hsi2-2* mutant, while *RAB18* mRNA levels were higher in *hsi2* than Col-0 following exposure to the ABA analog (Figure 8).

Of 6 genes tested that have been implicated in ABA signaling, mRNA levels for three (*ABF2*, *ABI2* and *HAB1*) [37] were highly induced by PBI425 but displayed identical expression patterns in Col-0 and *hsi2-2* (Figure 8). Transcripts for *PHOSPHOLIPASE Dα1* (*PLDα1*) and *ANAC019*, both positive regulators of ABA signalling [38,39], showed higher levels of expression in the mutant than wild-type following treatment with PBI425. With *PLDα1*, mRNA levels in *hsi2* were also higher in the control and PEG 8000 treatments, suggesting constitutive differences in expression rather than altered responses to ABA and drought. Expression of *ERA1*, which encodes a farnesyl transferase and a negative regulator of ABA signalling [40], was not inducible by PBI425 and showed no consistent differences between Col-0 and *hsi2-2*.

Two genes implicated in mediating drought stress (*CBF1* and *RD20*) were highly inducible by PBI425 (Figure 8). While *hsi2-2* and Col-0 displayed identical patterns of expression for *RD20* in control and PBI425 treatments, expression of the transcription factor *CBF1* was higher in *hsi2-2* following treatment with the ABA analog. Expression of neither *MYB15*, *MYB88* nor *ERECTA* were inducible by PBI425; however, levels of *MYB15* in *hsi2-2* were lower in the control, but not the PBI425 treatment and *MYB88* levels were higher in *hsi2* following PBI425 treatment. Levels of *ERECTA* mRNA also showed a trend of being higher in the *hsi2* mutant under different treatments, although differences were more obvious in the PEG 8000 treatment. Differences in *ERECTA* expression were also observed in rosette leaves before and after drought stress (Figure 8). Thus, the loss of HSI2 function did not affect the expression of most genes tested within 4 h of PBI425 treatment. A longer-term study may reveal latent changes in ABA-inducible expression, if any, of these genes in the mutant compared to the wild type.

## Discussion

Our analyses establish a role for HSI2 in response to water stress during the vegetative stage of the *Arabidopsis* life cycle. Specifically, loss of *HSI2* function results in plants better able to maintain physiological water potential under limiting water conditions, as indicated by reduced wilting and higher leaf RWC (Figure 2*)*, while overexpression of *HSI2* yields a complementary phenotype. These results are consistent with *HSI2* acting as a negative regulator. The ability of the *hsi2* mutant to avoid low water potential is likely attributed, at least in part, to lower stomatal conductance (Figure 4), effectively reducing transpirational water loss. Consistent with this phenotype, the mutant constitutively expresses higher levels of genes implicated in reducing water loss or improving water use. These include *ERECTA*, a positive regulator of water use efficiency through control of stomatal density and conductance [31] and *PHOSPHOLIPASE Dα1*, a positive regulator of ABA-induced stomatal movement and early-stage drought resistance [38] (Figure 8). Conversely, the transcription factor *GTL1*, a negative regulator of water use efficiency through its effect on stomatal density [32] is down-regulated in *hsi2* at stage 0. Despite these changes in gene expression, no differences in stomatal density were obvious between the *hsi2* mutants and their corresponding wild-types in preliminary analyses. Stomatal density in *Arabidopsis* is developmentally controlled and affected by the environment [41]. Accordingly, more detailed analyses are required of both stomatal density and aperture. It is noteworthy that *HSI2* transcripts are likely preferentially expressed in guard cells in the absence of stress [42,43], and thus could be involved in the direct or indirect regulation of guard cell genes.

An unanticipated finding of this study was the reduced levels of numerous metabolites and gene transcripts associated with tolerance to dehydration stress measured in the *hsi2-2* mutant, compared to Col-0. This includes lower levels of ABA and several ABA metabolites (Figure 5) as well as osmolytes and osmoprotectants (Figure 6). Global analysis of gene expression revealed that most genes up-regulated under severe drought conditions are expressed at lower levels in *hsi2-2* than in Col-0 (Additional file 3). Among GO terms highly enriched in these lists of genes include response to abiotic stimulus, response to water deprivation, and response to ABA stimulus (Table 2). It is noteworthy that levels of many of these metabolites continue to be induced, and levels of gene transcripts up-regulated, under drought conditions; it is the magnitude of change that is dampened in the mutant compared to the wild-type. These results are consistent with *hsi2-2* plants experiencing, or at least perceiving, a milder dehydration stress than the wild-type under the conditions tested. One possible explanation for these findings is that the ability of *hsi2-2* seedlings to maintain RWC and avoid low water potential for a longer time than Col-0 delays the onset of cellular dehydration and attenuates its severity. Indeed, based on our metabolite and gene expression data, there is no evidence to indicate that *hsi2-2* seedlings should have greater tolerance to dehydration stress. This includes expression analysis of several genes associated with dehydration stress in seedlings treated with a PEG solution that simulates dehydration stress (Figure 8). The possibility that HSI2 affects drought tolerance by regulating other, and possibly, novel pathways cannot be excluded at this time and requires further investigation. Among the GO terms most significantly enriched in genes differentially expressed in *hsi2* vs Col-0 are endomembranes and chloroplasts and represent potential targets for future studies. Included in the endomembranes group are a number of transporters (amino acid, water channel, polyamine transporters, and membrane) such as MATE efflux family proteins, proton-dependent oligopeptide transport (POT) family proteins, auxin efflux carriers, ABC transporters, a delta TIP (water channel protein), sugar and nitrate transporters and a cytochrome oxidase. Recently, differential expression of genes encoding pumps and transporters were associated with increased drought tolerance of rice near isogenic lines [13].

Seeds acquire desiccation tolerance during the final stages of maturation. Desiccation tolerance is also an important component of drought tolerance, and parallels between the two processes have been drawn previously [7]. HSI2 is known to be required for repression of seed maturation genes upon germination and the transition to vegetative growth [22,24,26]. In the absence of direct evidence for altered HSI2 activity in response of drought stress (Figure 1 and [23]), it could be argued that increased drought tolerance of the *hsi2* mutant is a secondary effect triggered by the derepression of seed desiccation genes during vegetative growth. Indeed, transcripts of four genes encoding LEA proteins are constitutively up-regulated in leaves of the *hsi2* mutant (Additional file 2) and GO terms consistent with antioxidant activity (e.g. flavonoids, oxidoreductases) are enriched (Table 2). However, these changes are unlikely to account for the altered dehydration avoidance responses described above. Overall, 23% of genes differentially regulated in *hsi2* at stage 0 are also differentially expressed during the later stages of seed maturation, with 16% showing the same directionality of change in both conditions (data not shown). These values are lower than those observed in seedlings of *hsi2* mutants [24,26]. Given that *HSI2* regulation of seed maturation genes is dependent on sugar [22], the differences are likely attributed to the inclusion of sucrose in the media used to grow seedlings. An alternative possibility is that HSI2 may only be required for a limited period of time during vegetative development for repressing seed maturation programs, as reported for PICKLE [44]. Of note, the seedling studies reported up-regulation of gene encoding master regulators of seed maturation (LEC1 and AFL clade B3 transcription factors). Of these, only transcripts of *FUS3* were detected above background in leaves three-week-old Col-0 or *hsi2* plants, and its expression was not altered by loss of *HSI2*. Thus, activation of seed maturation genes in leaves of the *hsi2* mutant is likely to involve different signalling events. In a number of organisms, genes regulating embryogenic events have been shown to be required at later stages of development, including in response to stress [45,46]. Thus, a role for HSI2 in repressing seed maturation genes upon germination and later, in response to water stress, is not unprecedented.

Our results indicate that HSI2 is not absolutely required for ABA signalling during drought stress. Both the (+)-ABA and its analog PBI425, which increase drought tolerance by activating ABA responses and ABA signalling [12,29], continued to effectively induce greater drought tolerance in both the *hsi2* mutant and *35S:HSI2* plants without altering the relative tolerance of these genotypes compared to the wild-type (Figure 3). PBI425 was also effective at reducing stomatal conductance in the mutant, with levels continuing to be lower than the wild-type following treatment (Figure 4). Furthermore, the expression of several genes involved in ABA synthesis and signalling was unaltered in the mutant 4 hr after treatment with PBI425 (Figure 8), suggesting the mutant is not affected in its ability to respond to this hyperactive ABA analog, and presumably to ABA itself. In cases where gene expression was different between the mutant and wild-type following PBI425, it was typically also altered in the absence of PBI425, suggesting an ABA-independent cause. Although differences in the levels of several ABA-responsive genes were observed between *hsi2* and wild-type when plants displayed visible wilting symptoms, these were measured several days following water withdrawal, thus complicating interpretation. Altered patterns of gene expression under these conditions are likely attributed, at least in part, to different levels of ABA in the mutant at wilting, which in turn we hypothesize to result for better maintenance of water potential in the mutant. Although the effects of PBI425 are mediated through ABA signalling, global transcript profiling indicates that the two compounds do not act in an identical fashion [29]. Furthermore, exogenous application of growth regulators is limited in its ability to resolve the function of endogenous ones. For these reasons, additional research will be required to study the relationship between HSI2 and ABA. This should include evaluating the role of endogenous ABA and ABA signalling by epistatic analysis of *hsi2* and mutants defective in ABA synthesis or perception, such as *nced3*.

Given that HSI2 is an EAR-dependent transcriptional repressor [23], it could act to limit the transcription of a subset of drought-inducible genes when water is abundant. Such target genes would be constitutively de-repressed upon loss of HSI2 function, as observed in comparisons of *hsi2* and Col-0 transcriptomes at stage 0 (Table 2 and Additional file 3), conferring a benefit to the mutant upon initial exposure to limiting water conditions. Several previously reported stress responsive genes from *Arabidopsis,* including *SFR6*, *LEA4-5*, *ATPAL1*, *AtCIMS*, *EGY3*, *ATCP1*, *ATHAL3*, *ATHSP-70*, *FAR1*, and *GDH1*, are differentially expressed in *hsi2* in the absence of a drought stress. Notably, nearly all of the differentially expressed genes involved in cellular/transmembrane transport (of sugar, lipids and metal ions) are up-regulated in the mutant suggesting more active transport and detoxification than in the wild-type. Also consistent with detoxification, possibly of reactive oxygen species known to accumulate in response to abiotic stress, is the enrichment of GO terms for oxidoreductases and flavonoids in *hsi2* at stage 0 (Table 2). We speculate that HSI2-mediated transcriptional repression is naturally relieved upon perception of limiting water conditions. Transcript levels of *HSI2* do not appear to be altered in response to stress (Figure 1 and [23]), suggesting that de-repression of drought-related HSI2 targets is likely mediated by translational or post-translational modification of HSI2. Several EAR-containing plant proteins have been shown to be post-translationally modified by phosphorylation or poly-ubiquitination, providing a potential means of relieving their repressive effects on gene expression (reviewed in [47]).

## Conclusions

By subjecting single *hsi2* mutants and plants overexpressing *HSI2* to simulated drought stress by withholding watering, we have demonstrated a role for the putative chromatin remodelling factor HSI2 during drought stress at vegetative stage of the *Arabidopsis* life cycle life. Although elucidating the exact role of HSI2 will require additional research, available information indicates that it fulfils a negative role in maintaining physiological water potential under limiting water conditions and as such represents a potential target for genetic manipulation towards the development of crops better suited for cultivation under water-limited environments. Formal demonstration that HSI2 activity is regulated in response to drought stress will be important to resolving its biological role at this stage of the plant life cycle. The identification of direct targets for HSI2, the dynamics of HSI2 binding to these targets and the associated epigenetic state of the targets following water withdrawal will be required to clarify the involvement and mechanism of action of *HSI2* in mediating drought-related gene expression.

## Methods

### Plant materials and growth conditions

*Arabidopsis thaliana* (L.) Heynh. was used throughout. T-DNA insertion lines, *hsi2-2* (SALK_088606) and *hsi2-5* (WiscDsLox388F10) were identified from the Salk Institute Genomic Laboratory Genomic database (http://signal.salk.edu; [48] and obtained from the *Arabidopsis* Biological Resource Centre (ABRC, http://abrc.osu.edu/). The locations of T-DNA insertions were confirmed by sequencing of PCR fragments and plants homozygous for the T-DNA insertions were identified by PCR (oligos used for PCR amplification are listed in Additional file 4).

To generate plants overexpressing *HSI2*, the genomic protein-coding region of At2g30470 was amplified by PCR from the BAC T6B20 (ABRC) and inserted sequentially into pDONRZeo (Invitrogen, http://www.invitrogen.com) and the pK7WG2 [49] derivative pER330 (E. Risseeuw and R. Datla, unpublished) using Gateway technology (Invitrogen). The integrity of the resulting *35S:HSI2* gene was confirmed by sequencing. Transgenic plants were generated by dipping inflorescences of Col-0 [50] in a suspension of *Agrobacterium tumefaciens* GV3101 (MP90) harboring the modified T-DNA binary plasmid and subjecting the resulting seeds to antibiotic selection (30 mg l^-1^ kanamycin). Independent T_3_ or T_4_ lines expressing the transgene were analyzed.

Unless otherwise noted, seeds of different genotypes were stratified for 2 days at 4°C, sown on Sunshine #4 potting mix (Sun Gro Horticulture, http://www.sungro.com/) and transferred to environment-controlled growth chambers with a 16-h photoperiod (200 μmol m^-2^ s^-1^) at 22/20°C (day/night). All samples for gene, hormone and metabolite analysis were collected at the same time of day to minimize circadian effects, immediately frozen in liquid nitrogen and stored at -80°C.

### Drought treatments and measurements

Plants were watered as needed to maintain the soil moisture near field capacity and fertilized weekly with 20 N:20P:20 K until three-week-old, at which point they were subjected to drought stress by withholding water. Visual wilting, indicated by progressive loss of lush green color of leaves and drooping of leaf blades and petiole, was monitored daily thereafter until more than 80% plants were wilting. At the end of the observation period, plants were re-watered to field capacity, and recovery was recorded 24 h thereafter. Drought response (visual wilting) and recovery from wilting upon re-watering were evaluated in three independent batches of plants, originating from at least 2 different seed lots, with 36–72 plants observed per batch. Leaf water status was measured when more than 50% of the plants started to wilt. Results are presented as leaf Relative Water Content (RWC = fresh weight-dry weight/turgid weight-dry weight) and as percentage of wilted or dead plants at, or over, a certain period of time. In experiments evaluating the effect of the ABA analog on the onset and progress of drought stress, three-week-old plants were subjected to root-dip treatment with 30 μM of (+)-ABA or 20 μM of the long-lived synthetic ABA analog, (+)-8’-acetylene ABA (PBI425) in 0.05% ethanol v/v [27] and watering was withheld thereafter. The concentrations of ABA and the PBI425 were chosen based on information from the published literature [12]. Preliminary dose–response experiments established these levels as being effective at delaying visual wilting in Col-0 without undesired side effects, such as the accumulation of anthocyanins and leaf curling, that were observed when higher concentrations of PBI425 or (+)-ABA were applied. Because the *hsi2* mutant responded very similarly to ABA and PBI425, and PBI425 offers several advantages over natural ABA when the sustained presence of hormonal activity is required, the synthetic analog was used in subsequent experiments. Student’s *t*-test (p ≤ 0.05) was performed to identify significant differences between treatments.

### Measurement of stomatal conductance

Stomatal conductance was measured from fully expanded young leaves from well-watered or PBI425-treated plants using a steady state leaf porometer (Model SC-1,Decagon Devices, http://www.decagon.com) following the manufacturer’s instructions. Porometer readings were taken from the adaxial side (lower surface) of leaves by placing the sensor head at the widest part of the leaf and holding it in place until the measurement was complete (~ 30 seconds/reading). Measurements were taken from 2–4 leaves per plant and 8–12 different plants from each genotype and treatment. Porometer readings taken different leaves of a plant were averaged to derive a single value, and genotype and treatment average were calculated thereafter. Measurements were performed between 9:30 am to 11:30 am, two h after lights were turned on in the cabinets.

### Quantification of ABA and metabolites by LC-MS/MS

Analysis was conducted at the National Research Council, Saskatoon, by UPLC-ESI-MS/MS (http://www.nrc-cnrc.gc.ca/eng/solutions/advisory/plant_hormone.html) as described in Chiwocha *et al.*[51]. For ABA profiling, tissues for analysis were harvested from the aerial portion of 3-week-old, well-watered (soil saturated to field capacity), drought-stressed (showing wilting) and re-watered (to field capacity, 24 h) plants. Each sample consisted of material pooled from six plants.

### Metabolomic profiling and analysis

For metabolic profiling analyses, tissues were harvested from the aerial portion of 3-week-old, well-watered (to soil saturation, near field capacity), drought-stressed (showing visible wilting) and re-watered (to soil saturation for 24 h) plants and frozen immediately in liquid nitrogen and stored at -80°C until analyzed. Samples were collected in three biological replicates per genotype and each sample (65–250 mg) consisted of material pooled from six plants. Frozen tissues were freeze-dried and analyzed by Metabolon Inc (http://www.metabolon.com) as described by Oliver *et al.*[52]. Briefly, samples were extracted and prepared for analysis using Metabolon’s standard solvent extraction method. The extracted samples were split into equal parts for analysis on the GC/MS and LC/MS/MS platforms. Also included were several technical replicate samples created from a homogeneous pool containing a small amount of all samples considered in the study. A total 156 named biochemicals were identified. Following log transformation and imputation with minimum observed values for each compound, ANOVA contrasts were used to identify biochemicals that differed significantly between two genotypes at various time points.

### Microarray analysis

Gene-expression profiles were generated using *Arabidopsis* 4 × 44 K oligonucleotide microarrays (http://www.agilent.com). Total RNA was isolated using the Qiagen RNAeasy Plant Mini Kit (http://www.qiagen.com) and quality was assessed using an Agilent-2100 Bioanalyzer. Each sample co-hybridized consisted of rosette leaves from four plants. Four independent biological replicates were analyzed at stage 0 (well-watered to field capacity) and three replicates at each of stage 1 (dry soil following withholding watering but before visible wilting) and stage 2 (visible wilting). Labeling and hybridization were performed following the manufacturer’s protocol using Agilent’s QuickAmp Labeling kit for two-color microarrays and incorporated a dye swap design. After washing, arrays were scanned and signals converted to expression data using GenePix 4000B scanner (GenePix Pro. 6.1, http://www.moleculardevices.com). Further in-house analysis was performed in GeneSpring GX.10 (Agilent). Additional analyses of differentially expressed genes were conducted using the following web-based tools; the Classification SuperViewer (The BAR; http://bar.utoronto.ca/ntools/cgi-bin/ntools_classification_superviewer.cgi), ATCOECIS (http://bioinformatics.psb.ugent.be/ATCOECIS), and Athena (http://www.bioinformatics2.wsu.edu/cgi-bin/Athena/cgi/analysis_select.pl). To compare expression levels of the wild-type before and after drought, single channel intensity values were obtained using the formula intensity calculator plugin in BASE 2.14.0 (http://base.thep.lu.se) and normalized with the Limma software package for R [53], using robust spline for intra-array and quantile for interarray normalizations. MIAME complaint data have been submitted to GEO (Accession # GSE39347).

### Kinetic polymerase chain reaction analysis

Total RNA was isolated using the Agilent Plant RNA Isolation Mini Kit and treated with amplification grade DNase I (Invitrogen). The first-strand cDNA was synthesized using SuperScript^®^ II reverse transcriptase (Invitrogen) and kinetic Polymerase Chain Reaction was performed on an MX3005P spectrofluorometric thermal cycler (Stratagene, http://www.stratagene.com) as described by Sharma *et al*. [54]. Amplification of the housekeeping gene encoding polypyrimidine tract-binding protein1 (PTBpa; AT3G01150) was used as control to normalize expression data. The list of genes analyzed and primers used in k-RT-PCR experiments are presented in Additional file 5.

For analysis of gene expression in response to PBI425 and polyethylene glycol, seeds were surface sterilized, stratified as above, and germinated on agar solidified 0.5× Murashige and Skoog (MS) medium (Sigma, http://www.sigma.com) in 150 × 15 mm Petri plates in a controlled environment chamber (80–100 μmol m^-2^ s^-1^ light Intensity, 16-h photoperiod, 24°C). Fourteen-day-old plants were flooded with PBI425 (25 μM) or 20% PEG 8000 on the germination plates and incubated at room temperature for 4 h prior to tissue harvest.

## Abbreviations

35S: Cauliflower mosaic virus 35S promoter; ABA: Abscisic acid; ABA-GE: ABA-glucose ester; ABI3: Absicic acid insensitive3; AFL: ABI3/FUS3/LEC2; DPA: Dihydrophaseic acid; FUS3: FUSCA3; GO: Gene ontology; HSI2: High-level expression of sugar inducible gene2; HSL1: HSI2-Like1; HSL2: HSI2-Like2; k-RT-PCR: Kinetic reverse-transcriptase PCR; LEA: Late-embryogenesis abundant; LEC2: Leafy cotyledon2; mDr: Moderate drought; OEx: Over expression (lines show increased levels of *HSI2* transcripts); PA: Phaseic acid; pDr: Progressive drought; PHD: Plant homeodomain; PLDα1: Phospholipase Dα1; RWC: Relative water content; t-ABA: *Trans*-ABA; VAL1: Viviparous ABI3-Like1.

## Competing interests

Patent applications have been filed on the use of HSI2 to enhance drought tolerance (International Patent Application PCT/CA2010/000754, United States Patent Application 13320813). Authors NS and PRF are inventors on these applications.

## Authors’ contributions

NS and PRF conceived the study, analyzed and interpreted the data, and drafted the manuscript with the assistance of KB and YB. NS and YB generated plant material and performed physiological studies. YB and KB performed gene expression analyses. KB assisted with microarray data analysis and with the preparation of figures and tables. All authors read and approved the final manuscript.

## Supplementary Material

Additional file 1 **Heat maps of metabolic responses in Col-0 and *hsi2-2 *** **to drought stress.** Click here for file

Additional file 2 **Annotated list of probe sets differentially regulated in leaves of *hsi2-2 *** **at each stages of drought.** Click here for file

Additional file 3 Summary of comparisons between genes found to be regulated by drought in the present study and the literature. Click here for file

Additional file 4 **Summary of comparisons between genes found to be regulated by drought in the present study and the literature, considering directions of expression changes.** Genes with >2.5× difference in average signal intensity between Col‒0 at stage 0 vs stage 2 were compared. Duplicate AGI codes were removed. Click here for file

Additional file 5 List of genes and primer sequences used in this study. Click here for file
