# Supplementary material for: High-level expression of sugar inducible gene2 (HSI2) is a negative regulator of drought stress tolerance in Arabidopsis
Source: BMC Plant Biol. 2013 Oct 29;13:170. doi: 10.1186/1471-2229-13-170 (PMC3893512; doi:10.1186/1471-2229-13-170)
Supplement: Additional file 5 — List of genes and primer sequences used in this study. [file 1471-2229-13-170-S5.doc]

Additional file 5

Primers used for characterization of T-DNA insertions:

*hsi2-2* (SALK_088606)

Forward: CCTCTTTCCAAAGACAGGAGG

Reverse: GGTGATGTCTTTTCTTGTCCTTG

T-DNA (LBb1): AACGTCCGCAATGTGTTATTAAGTTGTC

*hsi2-5* (CS854013, WiscDsLox388F10)

Forward: ACTCTCTTCTTTCTCCACCGC

Reverse: CCTCCTGTCTTTGGAAAGAGG

T-DNA (LB): GCGTGGACCGCTTGCTGCAACT

Primers used for k-RT-PCR:

| **Gene** | **AGI** | **Forward Primer Sequence** | **Reverse Primer Sequence** |
| --- | --- | --- | --- |
| *ABF2* | AT1G45249 | TTTGCAGGTTCAAGGTTCTAGTTTGCCTCT | TCGTTAAAGACTGATCTCCAAGTCCCACAA |
| *ABI2* | AT5G57050 | GTGTATTTGGTGTTCTCGCAATGTCAAGATCC | TTCGTTTGTCATTACATCCCAAAGACCATCAC |
| *ANAC019* | AT1G52890 | CATAATAACTCGGTACCGGAACTCGGAATG | TATTAAACCCGTGACTGCTCTCGACTTCCT |
| *CBF1* | AT4G25490 | CGATGGTGGAAGCTATTTATACACCGGAACAG | TTAGTAACTCCAAAGCGACACGTCACCATCTC |
| *COR78* | AT5G52310 | AAGCCTTTTCTGATATGGTTGCCGAGAAACTT | AACCATCCTTTAATCCTCCCAACCATTCCTC |
| *ERA1* | AT5G40280 | TTATCTGATGAAAGGCTTAAGGCAGCTTGG | TCCCCAAGCAAAGCTATTGAATGAAGAATC |
| *ERD6* | AT1G08930 | TGACGAATATTCACAGACATCTGTTCCATCCA | GCAGGGAACAGCTCTATAATCAGGCTTTGTTG |
| *ERECTA1* | AT2G26330 | TATTCCCTCTCTCACTTGGGGAGTCTTCTG | CTGCACCATATGCTATCTTAAGCCGTGTGT |
| *GTL1* | AT1G33240 | AACCGCCACAAGAAGGACTTGTTAATGTTC | GTCTTCTGGCTTTTCTGTTCCTTGTGGACT |
| *HAB1* | AT1G72770 | CGCGGGTGTAAATCAGGATACCAATACTTC | TGCCCCAAAGCGGTATACAATCTAGTTCAT |
| *KIN1* | AT5G15960 | ATGTCAGAGACCAACAAGAATGCCTTCCAA | CGGTCTTGTCCTTCACGAAGTTAACACCTC |
| *MYB15* | AT3G23250 | CGTATCGAATGACCTAGAAGTGGCTGGTTT | CCAATACATCGAACCAGAAGTCCATCTCAC |
| *MYB88* | AT2G02820 | CACTTTTGTGTGAGGCACAGAGATTGTTTG | TTATCCGTTCTTCCTGAGACCACTTTTGCT |
| *MYB96* | AT5G62470 | TTAACATGGCAAAACAAGCTCTTTGTGAGG | ATAACAACGGTCAAGCAAAGCTGATGAGAA |
| *PLDα1* | AT3G15730 | ATGTACAACTTCGAGCAGAGATGGAGCAAG | GTGGTCCTCTTGGAACATAACAGGAGAAGG |
| *RAB18* | AT1G43890 | GACAAGGAGGGAGGAGGAAGAAGGGAATAA | CAGACTGATCATGATGACCTGGCAACTTCT |
| *RD20* | AT2G33380 | ACCGAAGGAAGGTATGTCCCAGTTAACCTC | GATTTCCCTCGGTTACATTCCAAACCTCTT |
| *RD22* | AT5G25610 | GGTAAATATCACGTCAGGGCTGTTTCCACT | GTAGAACACCGCGAATGGGTACTTCTGTTT |
| *RD29B* | AT5G52300 | CAGAAGAACCAATCAGAATTCACCATCCAG | TCGTGATCATGACCATTTCCATGTTTAGTG |
| *HSI2* | AT2G30470 | GGAAGTGAACACAGCGAGAATAGACCTGAACA | CCGAACACTGTCCTATTGCTCTTTTTCGACTC |
| *PTB1* | AT3G01150 | AGAATATGCAGTATGCTGTCACTGTGGATG | TTGTCGCTAAAAGCCTTAACATTCAGATCA |
